# Use of Amulet in behavioral change for geriatric obesity management

**DOI:** 10.1177/2055207619858564

**Published:** 2019-06-21

**Authors:** John A Batsis, Alexandra B Zagaria, Ryan J Halter, George G Boateng, Patrick Proctor, Stephen J Bartels, David Kotz

**Affiliations:** 1 Section of General Internal Medicine, Dartmouth-Hitchcock Medical Center, United States of America; 2 Geisel School of Medicine at Dartmouth and The Dartmouth Institute for Health Policy & Clinical Practice, United States of America; 3 Dartmouth Centers for Health and Aging, Dartmouth College, United States of America; 4 Health Promotion Research Center at Dartmouth, United States of America; 5 Section of Weight & Wellness, Dartmouth-Hitchcock Medical Center, United States of America; 6 Thayer School of Engineering, Dartmouth College, United States of America; 7 Department of Surgery, Geisel School of Medicine, Dartmouth College, United States of America; 8 Department of Computer Science, Dartmouth College, United States of America

**Keywords:** Obesity, mHealth, Amulet, behavioral change, physical activity, sarcopenia

## Abstract

**Background:**

Obesity in older adults is a significant public health concern. Weight-loss interventions are known to improve physical function but risk the development of sarcopenia. Mobile health devices have the potential to augment existing interventions and, if designed accordingly, could improve one’s physical activity and strength in routine physical activity interventions.

**Methods and results:**

We present Amulet, a mobile health device that has the capability of engaging patients in physical activity. The purpose of this article is to discuss the development of applications that are tailored to older adults with obesity, with the intention to engage and improve their health.

**Conclusions:**

Using a team-science approach, Amulet has the potential, as an open-source mobile health device, to tailor activity interventions to older adults.

## Introduction

The obesity epidemic in the United States extends to adults over the age of 60 years with over 37% classified as having obesity.^1^ The consequences of obesity have been well established and extend beyond cardiometabolic risk^2^ and malignancy.^3^ Older adults are at high fall risk^4^ and can develop impairments that lead to a loss of independence.^5^ Intentional weight loss can improve physical function and quality of life.^6^ However, implementing behavioral management programs for obesity in primary care settings are logistically difficult due to busy, resource-limited infrastructures.^7,8^

Mobile health (mHealth) and remote sensor technologies are highly promising as an adjunct to health behavior-change interventions. Health behavior-change interventions augmented by mHealth may also include automated asynchronous, continual feedback to support patients in achieving their own personalized goals.^9–13^ Such feedback engages patients by providing knowledge, insight, and motivation in attaining nutritional, fitness, or other goals, by promoting disease self-management. The emergence of mHealth devices and platforms provides a mechanism for health-service interventions and for researchers to validate, evaluate, and assess their use with participants who otherwise have struggled with conventional therapeutic modalities in reaching their individualized goals.

A major barrier is usability of the technology for its target population.^14^ Employing a transdisciplinary-based user-centered design strategy helps ensure that technology can accommodate the specific needs and preferences of a target population. A population often ignored is older adults, who are the fastest-growing consumers of technology.^15^ In this example, we focus on older adults with obesity. Such an approach promotes the effectiveness of the device or tool that can integrate clinicians, researchers, usability experts, engineers, and computer scientists to effectively develop, validate, and deploy targeted mHealth interventions. By integrating different scientific approaches and viewpoints, there is the potential to solve complex multifaceted problems in mHealth research and maximize usability, implementation, and effectiveness outcomes in routine clinical settings. The purpose of this report is to describe how transdisciplinary care can specifically address the clinical challenges of obesity and sarcopenia in older adults.

### Amulet as an mHealth device

Amulet is an open-hardware, open-software, wrist-worn mHealth device developed by Dartmouth College and Clemson University.^16^ The process of designing and developing Amulet and subsequently adapting it for special clinical populations uses a transdisciplinary approach of team-science. The Amulet hardware has two microcontrollers: an MSP430 for running applications and an nRF51822 for communicating with peripheral Bluetooth Low Energy devices such as a heart-rate monitor or a galvanic skin response sensor. Its built-in sensors measure acceleration, rotation, ambient sound, ambient light, and ambient temperature. The main board has two buttons, capacitive touch sensors, a battery, a haptic buzzer, two LEDs embedded in the case, a secondary storage board that holds a microSD card reader, and a display screen. The energy efficiency of the Amulet system enables it to last weeks or months before needing to be recharged. The hardware designs and software of the latest model of the Amulet platform are available for download from GitHub. Amulet may be freely reproduced or modified for research and education purposes (see license on GitHub). In 2018, we manufactured 150 Amulets at an approximate cost of US$175 per unit. Amulet’s energy- and resource-efficient open-source platform allows the development of specific clinical applications to engage individuals in health behavior change. By using the capabilities of momentary assessment, Amulet can create and analyze information on the front end, moving beyond the end-user interface information that is normally the focus of consumers. This device uses low-energy Bluetooth 4.0 connection-oriented protocols, which allow seamless connections between Amulet and other devices or sensors. It also has the ability to produce and retain different data types and formats through minor software changes. Amulet’s hardware was developed to allow multiple applications to run concurrently and continuously without compromising battery life. This capability allows real-time monitoring of the physiological and behavioral health of its users.

In contrast, commercial devices such as the Apple Watch or Android Wear smartwatch have substantially limited battery life when running multiple applications, particularly during the collection of continuous data from internal and external devices and sensors. Devices such as Fitbit or Jawbone use proprietary software and algorithms preventing external researchers from performing validity and reliability testing. These limitations make these devices poorly suited for developing novel applications in research settings or for adapting them to specific populations. The open-source capability of the Amulet allows our research group to create and develop specific applications for use in older-adult behavioral studies. This is especially helpful in research pilot development of patient-specific applications. If created and deployed successfully, they can subsequently be adapted to other populations and refined accordingly.

Devices such as the ActiGraph, which is a research-grade, hip- or wrist-worn device, is often used to collect data about steps and activity from research participants.^17^ The ActiGraph collects raw accelerometry data that need extraction and processing offline using proprietary software. As a result, the ActiGraph cannot track steps and physical activity information in real time and cannot provide real-time feedback to subjects; with Amulet, this real-time feedback is a key aspect of our plan for obesity intervention. Also, the ActiGraph cannot be modified by researchers to perform real-time analysis of the data and potentially build some intelligence into this device.

Others have developed computationally efficient algorithms using tri-axial accelerometers (which they term Wockets) to classify four activity categories placed at the ankle and wrist: ambulation, cycling, sedentary, and other activities.^18^ As on the ActiGraph, real-time feedback is not provided. Also, their work focused on classifying activity groups without tracking the duration of time engaging in such activities. Importantly, though, placement of devices on the wrist is more likely to improve wear-time compliance, which is important for a system with the goal of improving physical activity among older adults.^18^

Our overall objective is to develop an application for integration into a multi-component 6-month wellness intervention tailored to the specific needs and capabilities of older adults with obesity. The physical activity component of this program engages participants in a 150-minutes per week aerobic activity program in addition to two 45- to 60-minute resistance exercise sessions.^19,20^ In applying the principles of transdisciplinary care to augmenting health behavior change, our group created three unique applications to monitor participants’ physical activity efforts and provide feedback: 1) a pedometer to count steps and measure distance traveled; 2) an activity monitor for activity type and duration; and 3) an interactive program for monitoring upper and lower extremity strength. Each application provides synchronous and asynchronous feedback through pre-programmed messages and goal-derived messaging to users.

### Why Amulet in an older adult population?

Older adults are the fasting-growing mHealth user group, yet it is a group whereby perceptions exist that using novel technologies may be problematic.^21^ Significant visual-sensory, dexterity, and cognitive processing issues are observed in this population, which also lead to potential usability issues that may not necessarily be problematic in younger age groups. The open-source Amulet provides an opportunity to tailor hardware and software design to the end-user. Although Amulet and our applications are still prototypes, the process of designing for a vulnerable, high-risk group provided our development team an opportunity to learn and surmount issues that may not be a problem in younger people. Furthermore, our ability to include multiple applications in a single device, and test them in controlled settings, will allow future modifications that ensure seamless deployment in the field.

Older adults cite various inconveniences with health technologies because of the physical and mental effort needed.^22^ We plan to address these concerns by involving older adults in our application-design process. For example, we plan to develop applications that require minimal user interaction and yet are fully functional. Also, these applications will be designed to run for long periods (such as weeks) before needing to be recharged, thereby reducing the burden of usage by older adults.

#### Pedometer application

The primary capacity of early wearable devices was to count steps. All major commercial devices (Fitbit, Garmin, Samsung Gear, Apple Watch) have algorithms that measure step counts. These devices measure the ability of users to reach pre-specified goals (normally ∼10,000 steps). However, closed-system devices do not provide access to step algorithms and step length to accurately calculate distance. Errors in accelerometry can potentially lead to inaccurate counts, thereby providing inaccurate data and misleading information in behavioral change both to clinicians and researchers. Although these devices are helpful in monitoring intra-individual variability, they are poor at discerning inter-person variability.^23,24^ Without the knowledge of proprietary algorithms, programmers are unable to improve their accuracy or modify them for target populations. Amulet’s pedometer application used a previously validated step-count algorithm from the literature that uses a wrist-mounted accelerometer.^25^

#### Activity application

**Figure 1.**
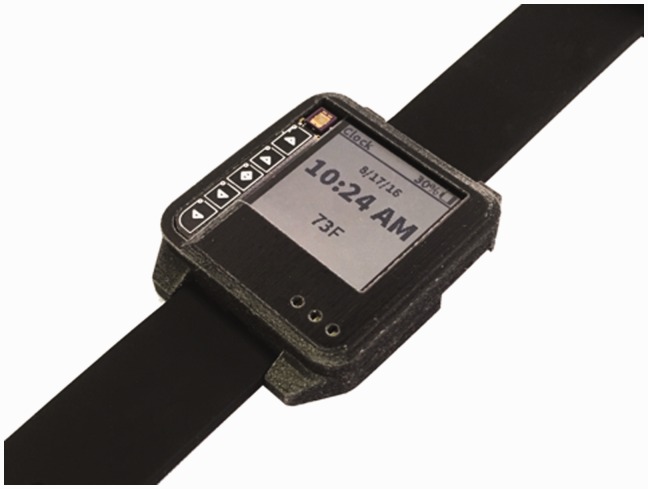
The Amulet prototype.

Cardiovascular fitness is an important predictor of longevity and functional decline. In our project, we applied a machine-learned support-vector-machine model to detect the activity level of individuals using Amulet.^26^ This application will allow continuous monitoring of activity-level data in real-time using acceleration data recorded from Amulet. The purpose of this application will be to use the recorded data to classify an individual’s activity level (see Figure 1). The Amulet will log data and display the results on the screen in an effort to motivate a patient to increase their activity level. Older adults will be involved in the design process for the activity display to ensure it is engaging for them. Information will ultimately provide feedback to the research team, who will then feed-forward this information back to the participant. Although this system has potential, we recognize it is common for device users to reduce their engagement over weeks or months.^27^ As such, our ultimate intent is that application data, in conjunction with real-time or asynchronous human feedback (electronically, video-conferencing, face-to-face, or by phone), can maintain subjects’ engagement in mHealth research. Applying user-centered design principles by incorporating the end user in design teams potentially can reduce attrition and disinterest in device use. The importance of appropriate stakeholders in product design in emerging technology cannot be overstated. This has the potential to display data that will be in a useful form to the target population.

**Figure 2.**
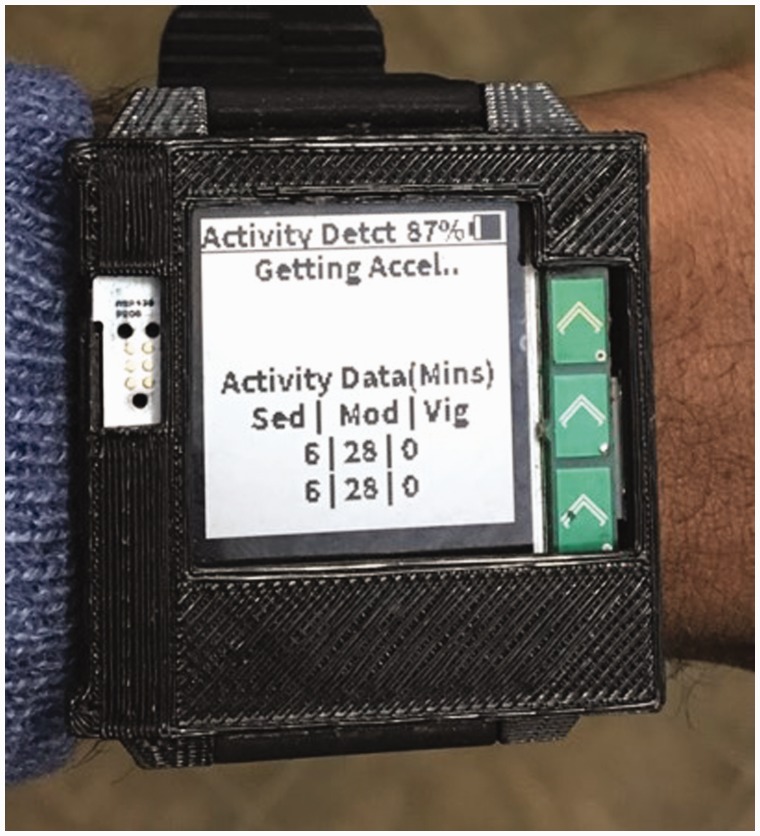
This application evaluates the activity of an individual and classifies it as low, moderate or vigorous.

The Amulet application will classify activity data as *sedentary, moderate,* or *vigorous,* in accordance with the Compendium of Physical Activities.^28^ The research team anticipates these physical activity data will be communicated to the research team via a secure cloud-based infrastructure, enabling the research team to provide meaningful feedback to the participant (Figure 2). Machine-learning scientists, exercise physiologists, therapist, clinicians, and application-developers are needed as part of this transdisciplinary team.

### Bluetooth-enabled resistance bands

**Figure 3.**
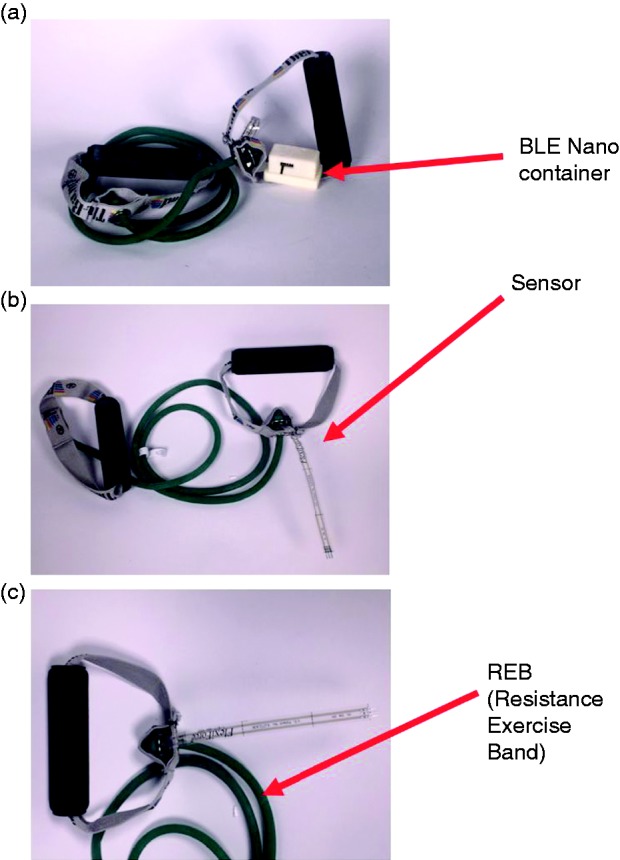
(a) The TheraBand low energy Bluetooth-Nano sensor system from the front; (b) from the side angle; (c) the sensor/handle interface.

**Figure 4.**
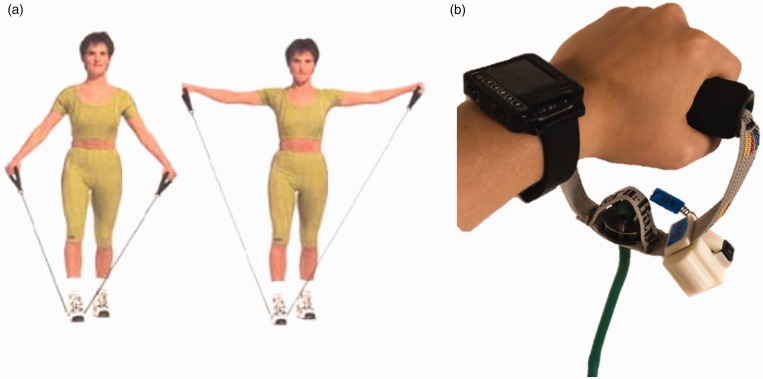
(a) A typical exercise that can be conducted using a resistance band; (b) Amulet with a resistance band.

Resistance training is an under-recognized element of physical activity programs in older adults.^29,30^ Resistance bands and free weights support helpful exercises, which can lead to muscle protein synthesis and hypertrophy, neuromuscular modulation, and enhancement of muscle mass and strength.^6,31,32^ In turn, these biological processes can mitigate age-related sarcopenia. Our goal is to create a device that participants could use in their home environment and provide real-time data on force and strength. A modified resistance-band apparatus, designed by a team of engineers at Dartmouth College, consists of resistance-band tubing fastened to plastic handles attached to an Arduino device that can communicate via a wireless Bluetooth connection to Amulet (Figure 3). This application will include a graphical interface providing individuals with the current, daily mean, and previous force/strengths as encouragement to continue their exercises. In Figure 4 we present a schematic of a specific exercise is conducted using a resistance band, with other properties described elsewhere (Batsis, in press).^33^

### Importance of Amulet in activity monitoring

Unfortunately, traditional methods of engaging older adults in physical activity have only been marginally successful.^34^ Although efficacy trials have demonstrated that both aerobic and resistance training are helpful in improving physical performance and lean mass,^6,29^ implementing these interventions in community-based settings has been difficult.^35^ Prevention of weight-loss-induced sarcopenia is often overlooked by practitioners, and physical activity monitoring of motivated older adults with obesity has the potential to mitigate the loss of muscle mass and strength that could exacerbate functional decline. The ability to oversee, monitor, and promote self-management provides an additional approach to motivate change in this population. The adaptable Amulet mHealth platform provides numerous additional opportunities for developing novel applications that can be adapted to the specific needs of special populations. The applications in development have the potential to be part of an integrated platform that simultaneously communicates with office staff and other informatics-based systems such as electronic medical records.

## Conclusions

mHealth devices are an emerging technology with the potential to promote positive behavior change. Commercial devices, although useful, lack in validity, openness, and reliability for clinical research purposes. Tailored mobile applications can be clinically effective in providing activity and obesity interventions for older adults. We used a transdisciplinary, team-science approach to overcome some of the challenges in the mHealth space, working collaboratively in the pilot testing of several mHealth applications by bringing together clinical and computing researchers on the Amulet platform. This novel platform can be adapted to target activities of special populations and enable clinicians and technology developers to work together to address challenging health behaviors. Successful deployment, validation, and integration into clinical-care systems are the next steps in evaluating the potential of this approach to achieve clinically significant improved outcomes in vulnerable, complex patient populations like older adults.
